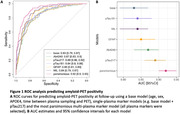# Predictive accuracy of plasma markers for Alzheimer’s disease, cerebrovascular disease, and cognition in a population‐based study

**DOI:** 10.1002/alz.091056

**Published:** 2025-01-09

**Authors:** Julia Neitzel, Anna M Streiber, Yuan Ma, Phuong Thuy Nguyen Ho, Frank J. Wolters, Albert Hofman, Daniel Bos, Meike W. Vernooij

**Affiliations:** ^1^ Erasmus University Medical Center, Rotterdam Netherlands; ^2^ Harvard T.H. Chan School of Public Health, Boston, MA USA

## Abstract

**Background:**

Validating the predictive value of plasma markers for Alzheimer’s disease, cerebrovascular disease and cognitive dysfunction in older adults representative of primary care settings, is needed to guide prevention and treatment decisions.

**Method:**

At baseline, single molecule array plasma measures of Aβ42/40, pTau217, pTau181, GFAP and NfL were collected from 625 dementia‐free participants from the population‐based Rotterdam Study (mean age 63 years [range 54‐84], 51% women). After a mean follow‐up time of seven years [range 5‐9], participants underwent amyloid ^18^F‐florbetaben PET, brain MRI, and cognitive assessment. We investigated cross‐sectional associations between basic sample characteristics and plasma markers using linear/logistic regression. We estimated how accurately single plasma markers at baseline predicted amyloid‐PET positivity, high white matter hyperintensity burden (i.e. volume in 4^th^ quartile) and low cognitive performance at follow‐up (i.e. composite score in 1^st^ quartile) using receiver operating characteristic curve analysis. Predictive performance between different models was compared with DeLong test. We further identified the most parsimonious multi‐plasma marker model based on the lowest Akaike information criterion.

**Result:**

Older participants had more abnormal plasma markers. Women showed higher GFAP, but lower pTau217 and 181 levels than men. APOE4 carriership was associated with more abnormal Ab42/40 and pTau217 levels. Education was not related to plasma markers. At follow‐up, 102 participants (16%) were amyloid‐PET positive. The area under the curve (AUC) for amyloid‐PET positivity using a base model containing age, sex, APOE4, and follow‐up time was 0.83 (Figure 1). Predictive accuracy significantly improved upon adding to the base model either GFAP (AUC=0.85), Aβ42/40 (AUC=0.87), or pTau217 (AUC=0.89; all p[DeLong]<0.05; Figure 1). AUC improved further to 0.92 for the most parsimonious model including all plasma markers (p[DeLong]<0.001). Using cutoffs based on Youden’s index, we estimated that 6.2 PET scans were needed to obtain one amyloid‐PET positive individual after screening with the base model. This number decreased to 4.7 or 2.8 when pTau217 or all plasma markers were considered. No plasma marker model seemed to outperform the base model for predicting high white matter hyperintensities or low cognitive performance.

**Conclusion:**

Plasma pTau217, Aβ42/40 and GFAP levels markedly improved the prediction of amyloid‐PET positivity in the general population